# Clinician-Recorded Temporomandibular Disorder at 12 Months After Digitally Planned Mandibular Fracture Repair: Associations with Condylar Injury and Early Functional Signs

**DOI:** 10.3390/jcm15166426

**Published:** 2026-08-20

**Authors:** Yifan Chi, Fei Xie, Jun Hou, Yukun Hu, Honghao Wang

**Affiliations:** 1 Department of Stomatology, The First Affiliated Hospital of Anhui Medical University, Hefei 230022, China; evachi@fy.ahmu.edu.cn (Y.C.); fxiekq@126.com (F.X.); houjun@ahmu.edu.cn (J.H.); yfy152725@fy.ahmu.edu.cn (Y.H.); 2 Department of Stomatology, The Second Affiliated Hospital of Anhui Medical University, Hefei 230601, China

**Keywords:** mandibular fracture, condylar fracture, digital surgical planning, temporomandibular disorder, malocclusion

## Abstract

**Background/Objectives:** Functional outcomes after digitally planned mandibular fracture repair remain incompletely characterized. This study estimated the 12-month frequency of clinician-recorded temporomandibular disorder (TMD) and examined its associations with condylar injury and clinical findings documented at 3 months. **Methods:** This single-center retrospective cohort included patients treated between September 2021 and June 2023. Of 139 patients eligible after criterion review, 18 (12.9%) lacked an observed 12-month outcome and 121 were analyzed. The endpoint was the treating-team clinical diagnosis after structured symptom review and examination; preserved records did not support full Diagnostic Criteria for Temporomandibular Disorders (DC/TMD) Axis I decision rules, subtype assignment, or Axis II instruments. Associations with condylar involvement were estimated using modified-Poisson regression with robust variance; the adjusted model included age, sex, and displacement grade. Associations with 3-month findings were exploratory and multiplicity-controlled. **Results:** Thirty-three of 121 patients had clinician-recorded TMD at 12 months (27.3%; 95% confidence interval [CI], 20.1–35.8%). TMD occurred in 27 of 47 patients with condylar involvement (57.4%) and 6 of 74 without it (8.1%; risk ratio [RR], 7.09; 95% CI, 3.17–15.86). The adjusted RR was 6.87 (95% CI, 3.06–15.41), with similar results when displacement grade was categorical. Malocclusion, TMJ clicking, TMJ pain, abnormal opening pattern, limited mouth opening, and unilateral chewing at 3 months remained associated after correction (all q ≤ 0.003). **Conclusions:** Clinician-recorded TMD was concentrated among patients with condylar injury. Associations with early functional abnormalities may reflect persistence or recurrence because several findings overlap with the later endpoint. These data support closer functional surveillance but do not establish causality, independent prediction, or the comparative effectiveness of digital planning.

## 1. Introduction

Large retrospective and multicenter trauma cohorts show that maxillofacial fractures occur in heterogeneous patterns and may be accompanied by dental and soft-tissue injuries [1,2,3]. Beyond restoring mandibular continuity and contour, treatment must also address clinically important functional outcomes, including stable occlusion, comfortable jaw movement, and masticatory recovery.

Digital surgical planning is increasingly used in maxillofacial trauma. An accuracy study in maxillofacial-fracture models described a digital occlusal relationship reconstruction workflow [4]. Digitally assisted planning may additionally incorporate three-dimensional reconstruction, virtual reduction, mirror-based planning, printed models, patient-specific guides, and precontoured fixation. These technologies comprise configurable tools rather than a single uniform intervention; their patient-level components require explicit reporting when comparative effects are evaluated. However, most published work has emphasized technical accuracy and bony alignment, whereas postoperative TMJ symptoms and functional recovery have received less attention.

TMDs comprise pain-related and intra-articular conditions involving the TMJ, masticatory muscles, and associated structures. Protocolized DC/TMD assessment combines an Axis I symptom history and defined clinical examination with Axis II instruments for pain-related disability, psychological distress, oral behaviors, and jaw functional limitation [5,6]. This dual-axis structure separates clinical diagnosis from patient-reported functional and psychosocial impact. Acute condylar injury can be accompanied by disk, capsular, and other periarticular soft-tissue abnormalities [7], and the OPPERA cohort found a strong association between incident injury and subsequent incident TMD [8]. Broader etiologic models also recognize interacting biological, behavioral, and psychosocial contributors [6]. Population estimates vary substantially, and a recent meta-analysis reported an overall prevalence of approximately 34% [9]. Postoperative TMD has also been documented after repair of mandibular fractures not involving the condyle [10].

Injury morphology is particularly relevant after mandibular trauma. Condylar fractures directly involve the functional articulation and can be accompanied by acute intra-articular soft-tissue abnormalities [7]. In broader mandibular-fracture cohorts, displacement and other injury-severity features have been associated with general postoperative complications [11,12]; these outcomes are not equivalent to TMD. Broad categories such as mandible-only or panfacial injury may therefore be less informative than joint-specific anatomy.

The present study was not designed to compare digital planning with conventional surgery. It describes the 12-month frequency of clinician-recorded TMD within a digitally planned cohort and evaluates its association with condylar injury. It also explores longitudinal associations between findings documented at 3 months and the later endpoint. Because several early findings are themselves manifestations considered during TMD assessment, they are treated as clinical correlates or persistence signals rather than independent predictors.

The objectives were to estimate the frequency of clinician-recorded TMD at 12 months after digitally planned mandibular fracture repair, examine associations with condylar and other recorded injury characteristics, and quantify exploratory longitudinal associations between 3-month clinical findings and the 12-month outcome.

## 2. Materials and Methods

### 2.1. Study Design, Setting, and Participants

This single-center retrospective cohort included consecutive patients with maxillofacial fractures involving the mandible who were treated at the First Affiliated Hospital of Anhui Medical University between September 2021 and June 2023. All included patients underwent digitally planned mandibular fracture repair. The study was designed as a postoperative outcome study, not as a comparison of digital and conventional treatment, and is reported with reference to the Strengthening the Reporting of Observational Studies in Epidemiology recommendations [13].

Inclusion criteria were age 16–65 years; a fracture line involving the mandible; use of digital planning during fracture repair; complete clinical and imaging records; and occlusal information sufficient for postoperative assessment. Exclusion criteria applied during criterion review were inability to assess occlusion; edentulism without a reliable preinjury occlusal reference; use of bone-metabolism-modifying medication; multiple severe systemic comorbidities; previous orthodontic treatment that impaired occlusal interpretation; severe neuromuscular disease affecting jaw function; and documented treatment for TMD or persistent TMJ symptoms before injury. Eligible patients without an observed 12-month outcome were retained in the participant-flow denominator but did not enter the outcome analysis.

**Figure 1:**
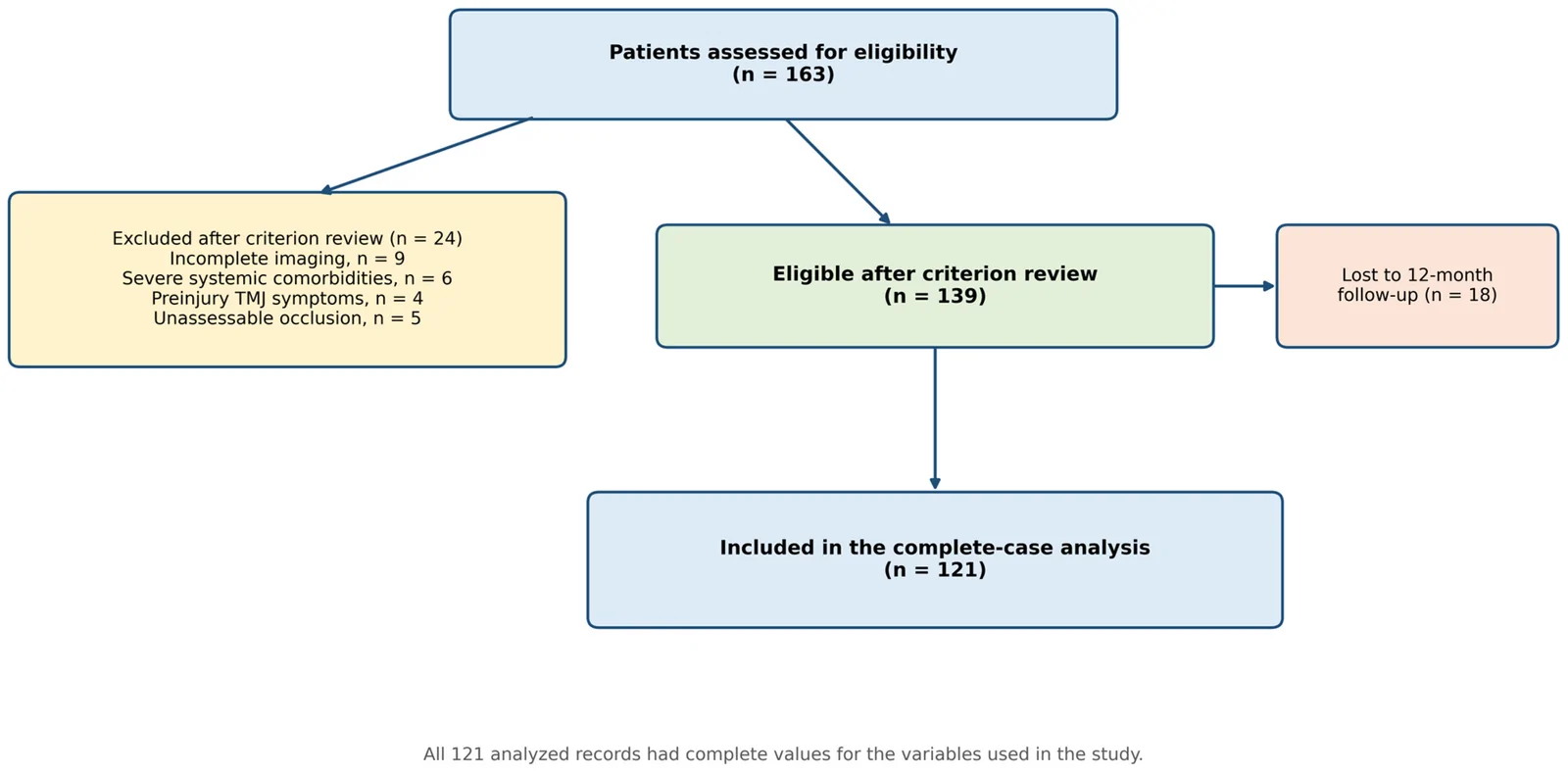
Participant flow diagram. Of 163 patients assessed for eligibility, 24 were excluded after criterion review. Of 139 eligible patients, 18 (12.9%) were lost before the 12-month assessment, yielding an analytic cohort of 121 patients with an observed primary outcome.

Of 163 patients assessed for eligibility, 24 were excluded after criterion review: 9 had incomplete imaging records, 6 had severe systemic comorbidities, 4 had documented preinjury TMJ symptoms, and 5 had unassessable occlusion. Of the remaining 139 eligible patients, 18 (12.9%) were lost before the 12-month assessment, leaving 121 patients with an observed primary outcome for analysis (Figure 1). Patient-level baseline information for the 18 lost patients was not preserved in the analysis file; baseline comparisons with retained patients and exposure-specific attrition analyses could therefore not be performed.

The study was approved by the Clinical Ethics Committee of the First Affiliated Hospital of Anhui Medical University (Approval No. Quick-PJ 2021-10-27) and was conducted in accordance with the Declaration of Helsinki. Written informed consent was obtained in accordance with the approval and institutional requirements.

### 2.2. Digital Planning and Perioperative Care

**Figure 2:**
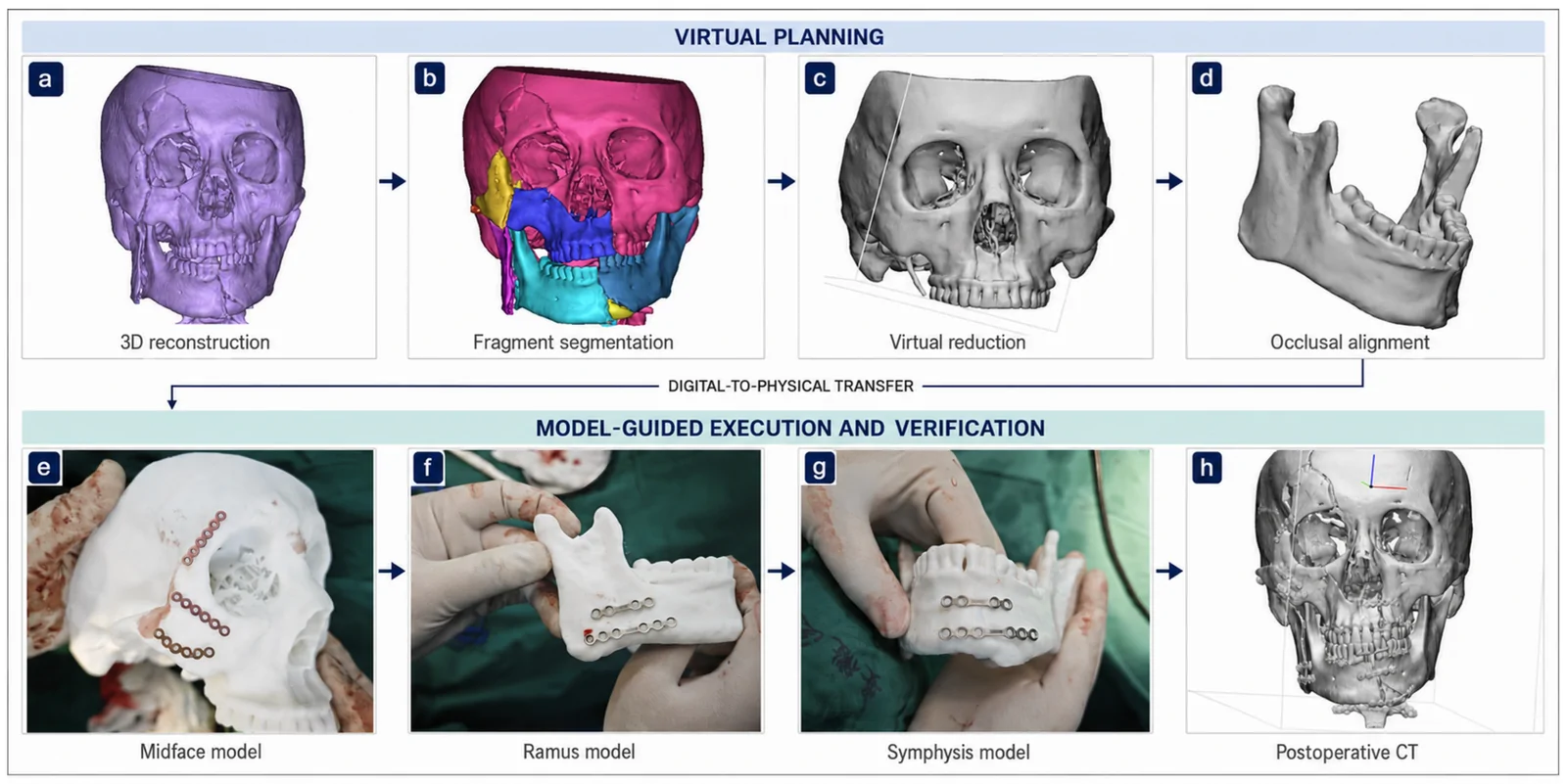
Integrated digitally assisted treatment workflow in a representative patient with complex maxillofacial fractures. (**a**) Preoperative CT-based three-dimensional reconstruction; (**b**) color-coded segmentation of major fracture fragments; (**c**) virtual midfacial reconstruction after reduction; (**d**) virtual mandibular reduction with occlusal alignment; (**e**) 3D-printed midfacial model with prebent fixation plates; (**f**) 3D-printed mandibular ramus model with prebent plates; (**g**) 3D-printed symphyseal model with prebent plates; and (**h**) postoperative CT verification of reduction and fixation. This is a representative case-based illustration of the available workflow, not a standardized protocol applied to the entire cohort. Image-based reconstruction and virtual reduction were the shared minimum; printed models, prebent plates, and other optional components were not used in every patient.

Preoperative CT or CBCT data were imported into Mimics 21.0 (Materialise, Leuven, Belgium Materialize, Belgium) for segmentation and three-dimensional reconstruction(Figure 2a,b). Models were exported in STL format and refined in 3-Matic (Materialise, Leuven, Belgium). Virtual reduction and, when clinically appropriate, mirror-based reconstruction using the contralateral side were performed before fixation planning (Figure 2c,d). The minimum shared workflow comprised image-based reconstruction, virtual fracture reduction, and production of a physical or virtual reference for intraoperative reduction. Depending on fracture morphology and clinical requirements, cases could additionally use printed models, patient-specific guides, precontoured fixation, or navigation(Figure 2e–h). Standardized patient-level counts for these optional components were not preserved; individual digital techniques were therefore neither analyzed as exposures nor compared. The source materials did not support retrospective assignment to reproducible digital or operative subgroups. Operative reduction, fixation approach, and optional digital adjuncts were individualized by fracture pattern; consequently, “digitally planned” denotes the cohort’s treatment context rather than a uniform operative protocol.

Standard postoperative care included anti-inflammatory treatment for 3 days, intermaxillary elastic traction or fixation for 1–2 weeks when clinically indicated, and progressive mouth-opening exercises with a target maximal interincisal opening of at least 35 mm by 4–6 weeks. Follow-up was scheduled at approximately 1, 3, and 12 months. The analysis dataset did not retain drug and dose details, side-specific condylar management, open versus closed treatment of condylar fractures, operative approach, fixation construct, or standardized rehabilitation adherence. TMD-specific physiotherapy, occlusal adjustment, splint therapy, and other individualized interventions were provided when clinically indicated but were not systematically retained.

### 2.3. Assessment Schedule and Data Sources

Preinjury information was obtained from available records and structured clinical history. The 1-month visit focused on wound healing, occlusal stability, mouth-opening range, and early TMJ symptoms. Candidate clinical findings were recorded primarily at the 3-month visit, and the primary endpoint was determined at 12 months. Variables were abstracted from clinical, imaging, and operative records into a de-identified dataset. The preserved study materials did not permit reconstruction of duplicate masked abstraction, independent morphology re-review, or formal interobserver reliability assessment.

### 2.4. Twelve-Month TMD Endpoint

The primary endpoint was the presence of clinician-recorded TMD at 12 months, as determined by the treating surgical team. The clinical assessment included structured history for jaw or temple pain, pain modified by jaw function, joint noise, locking, chewing difficulty, and parafunctional behavior; measurement of maximal interincisal opening; observation of opening and closing trajectories; palpation of the TMJ and masticatory muscles when clinically indicated; and assessment of TMJ sounds during mandibular movement.

The assessment was informed by DC/TMD principles [5,6], but the retrospective records did not preserve item-level decision rules, the minimum combination of findings required for a positive label, subtype-specific diagnoses, systematic magnetic resonance imaging, examiner calibration, or examiner blinding to injury morphology and earlier findings. The endpoint should therefore be interpreted as a treating-team clinical diagnosis rather than a protocolized DC/TMD diagnosis. Neither a complete Axis I protocol nor Axis II instruments could be reconstructed from the preserved records; protocolized scores for pain-related disability, psychological distress, oral behaviors, and jaw functional limitation were unavailable. Pain-related and intra-articular subtypes could not be analyzed separately, and outcome misclassification cannot be excluded.

Preinjury TMD was excluded through review of available records and patient history concerning persistent TMJ pain, recurrent joint noise, previous TMJ treatment, locking, or preinjury restriction or deviation of mouth opening. Mild or intermittent preinjury manifestations may have been missed because the study did not include a prospective preinjury examination.

### 2.5. Injury Morphology

The supplemented chart abstraction recorded condylar involvement as present or absent. When present, condylar level was categorized as condylar head (intracapsular), condylar neck, or subcondylar/base. The dataset also retained one mandibular fracture-site code per patient (symphysis/parasymphysis, body, angle, ramus, condyle, or coronoid), overall fracture laterality (unilateral or bilateral), clinician-recorded displacement grade (none, mild, or marked), comminution, and open-fracture status.

Multiple mandibular sites can coexist, but the retrospective dataset retained only one mandibular site code and did not preserve the rule used to select that code. Site-specific results were consequently treated as descriptive and were not entered into the adjusted model. Quantitative thresholds for displacement categories, the operational definition of open fracture, side-specific condylar level, and morphology-reviewer calibration were also not preserved.

### 2.6. Definitions of Early Clinical Signs

Malocclusion was defined as a clinically evident discrepancy from the planned or premorbid occlusal relationship, including open bite, crossbite, premature contact, occlusal midline deviation with interference, or patient-reported bite instability confirmed clinically. TMJ clicking was reproducible audible or palpable clicking during opening, closing, or excursive movement. TMJ pain was preauricular or intra-articular pain during jaw function or clinical examination. Limited mouth opening was maximal interincisal opening below 35 mm. Abnormal mouth-opening pattern comprised visible deviation, deflection, S-shaped movement, or a non-smooth opening or closing trajectory.

Unilateral chewing was clinician-recorded habitual and persistent use of one side during eating, based on structured enquiry and observation when available. Continuous laterality percentages were not preserved. Bruxism and clenching were based on patient or caregiver report. Postoperative anxiety represented clinician-recorded or patient-reported anxiety considered relevant to jaw function or rehabilitation. Deep overbite was a preinjury anatomical characteristic and was interpreted separately from postoperative findings. Infection and non-union were defined from clinician-recorded postoperative diagnoses and management; no non-union occurred.

### 2.7. Statistical Analysis

The primary analysis included the 121 patients with an observed 12-month outcome; no value was missing for the variables used, and no imputation was performed. Continuous data are presented as mean ± standard deviation and categorical data as counts and percentages. Age was compared using the Welch independent-sample *t* test. Pearson chi-square tests were used for sex, broad fracture region, fracture etiology, laterality, and displacement grade; the remaining testable binary characteristics were compared using two-sided Fisher exact tests. Condylar level and the single recorded mandibular site were compared using Monte Carlo permutation tests with 200,000 permutations because of sparse cells. Overall and subgroup risks are reported with Wilson 95% confidence intervals (CIs).

For binary characteristics and 3-month findings, absolute risks and unadjusted risk ratios (RRs) with log-method 95% CIs were calculated. The association of condylar involvement was quantified using modified-Poisson regression with HC0 robust sandwich variance [14]. The limited primary model adjusted for age per 10 years, sex, and displacement grade treated as an ordinal injury-severity marker. These covariates were selected before outcome modeling to limit model complexity with only 33 events. Additional adjustment for open-fracture status, comminution, and other clinically relevant injury characteristics would have increased the parameter burden and risked overfitting and unstable estimates. Because an ordinal coding assumes equal increments between displacement categories, a sensitivity model represented mild and marked displacement with separate indicator variables. The models were intentionally parsimonious and explanatory rather than exhaustive adjustment or prediction models. Three-month findings were not entered into an adjusted prediction model because they were measured after treatment and several overlapped with manifestations used to determine the 12-month endpoint.

The multiplicity family comprised 11 testable exploratory variables: limited mouth opening, TMJ clicking, TMJ pain, abnormal opening pattern, infection, malocclusion, bruxism, unilateral chewing, postoperative anxiety, clenching, and deep overbite. Non-union was excluded because all 121 values were zero. Two-sided Fisher exact *p* values were adjusted using the Benjamini–Hochberg procedure; q < 0.05 indicated that an association persisted after multiplicity control. Deep overbite was included in the correction family but interpreted separately because it was a baseline anatomical characteristic. To minimize selective reporting, complete results for all 11 variables—including nonsignificant findings—are reported in Supplementary Table S1. To assess the possible effect of missing 12-month outcomes on the overall frequency, extreme-case bounds assumed that all 18 lost patients either did not or did have TMD; these bounds were not treated as imputed estimates and do not address exposure-specific attrition. Analyses were reproduced in Python 3.12.13 using NumPy 2.3.5, pandas 2.2.3, and SciPy 1.17.0. All tests were two-sided.

## 3. Results

### 3.1. Cohort Characteristics and Data Completeness

All 121 analyzed records were complete for the variables used. Age ranged from 17 to 64 years, with a mean of 39.6 ± 13.6 years; 84 patients (69.4%) were male, and no non-union occurred. Demographic and injury characteristics by 12-month outcome are summarized in Table 1. Broad fracture region and injury etiology did not differ between outcome groups.

At 12 months, 33 patients had clinician-recorded TMD (27.3%; 95% CI, 20.1–35.8%) and 88 did not. If the 18 lost patients were added to the denominator, the overall frequency would be 23.7% if none had TMD and 36.7% if all had TMD. These are extreme-case bounds for the overall frequency, and not imputed estimates or corrections for exposure-specific attrition.

### 3.2. Injury Morphology and Twelve-Month TMD

Condylar involvement was present in 47 patients (38.8%). TMD occurred in 27 of 47 patients with condylar involvement (57.4%) and 6 of 74 without condylar involvement (8.1%), corresponding to an unadjusted RR of 7.09 (95% CI, 3.17–15.86; Fisher exact *p* < 0.001). In the limited modified-Poisson model adjusted for age, sex, and ordinal displacement grade, condylar involvement remained associated with the outcome (adjusted RR, 6.87; 95% CI, 3.06–15.41; *p* < 0.001). The estimate was similar when displacement grade was represented categorically (adjusted RR, 7.34; 95% CI, 3.41–15.78; *p* < 0.001); complete model estimates are presented in Supplementary Table S2.

**Figure 3:**
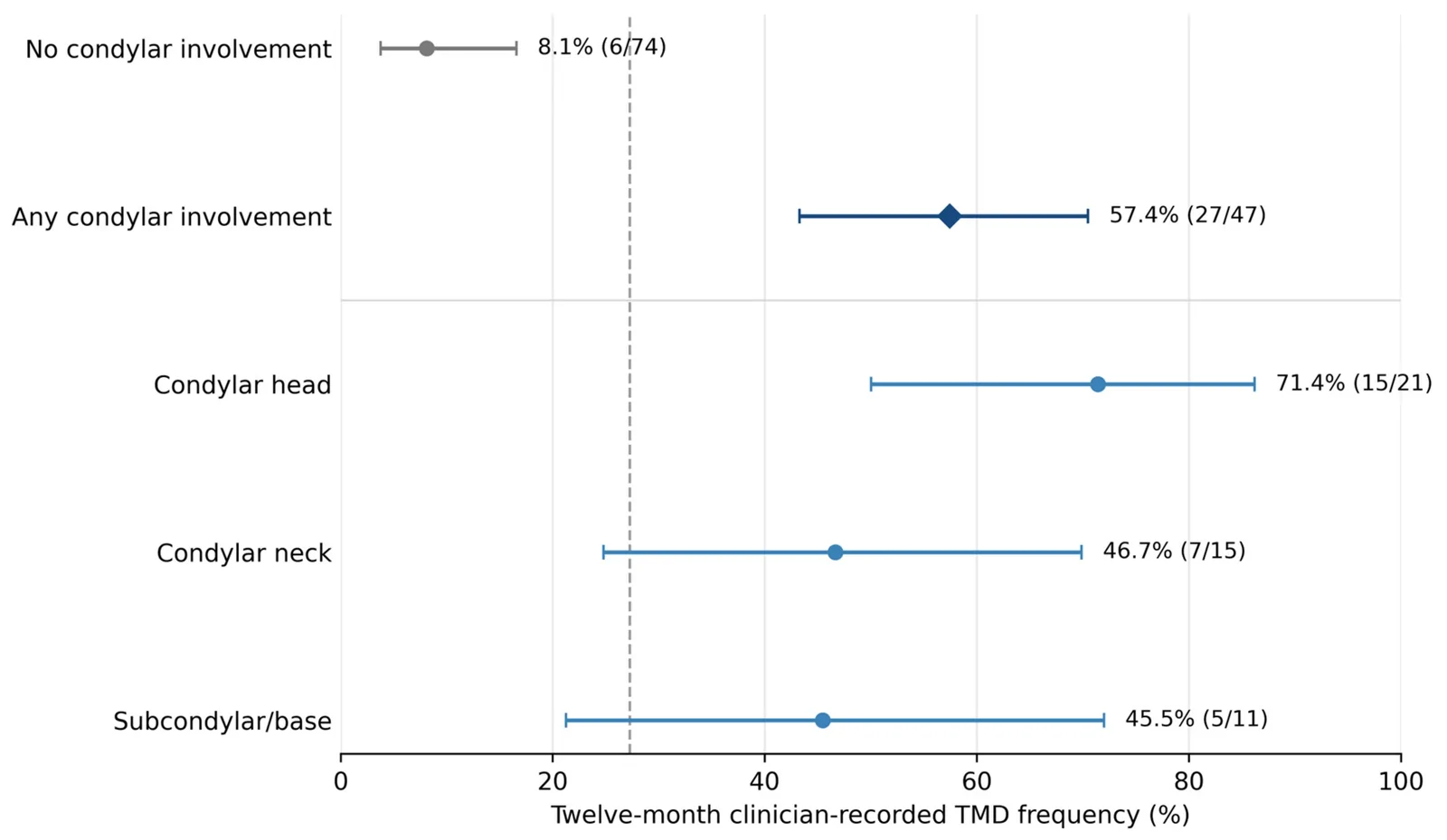
Absolute frequency of clinician-recorded TMD at 12 months by condylar involvement and fracture level. Points indicate observed risks and horizontal bars indicate Wilson 95% CIs; labels show percentages and case counts. The diamond denotes the prespecified aggregate group with any condylar involvement, indented rows show descriptive level-specific estimates, and the dashed line marks the overall cohort frequency (27.3%). The across-level Monte Carlo permutation test yielded *p* < 0.001. (The gray dashed vertical line indicates the overall 12-month clinician-recorded TMD frequency in the study cohort).

Figure 3 shows the prespecified contrast between any and no condylar involvement together with descriptive fracture-level estimates. The highest point estimate occurred after condylar-head fracture, although the subgroup CIs were wide; the across-level comparison was significant (Monte Carlo *p* < 0.001). Because level-specific sample sizes were small, these estimates are descriptive and should not be used to rank condylar levels. The single recorded mandibular-site code was also associated with the endpoint but was interpreted descriptively because multi-site injuries had been compressed into one code. Laterality, displacement grade, comminution, and open-fracture status were not associated with the endpoint in univariable comparisons (Table 1).

### 3.3. Associations of Three-Month Clinical Signs with the Twelve-Month Outcome

**Figure 4:**
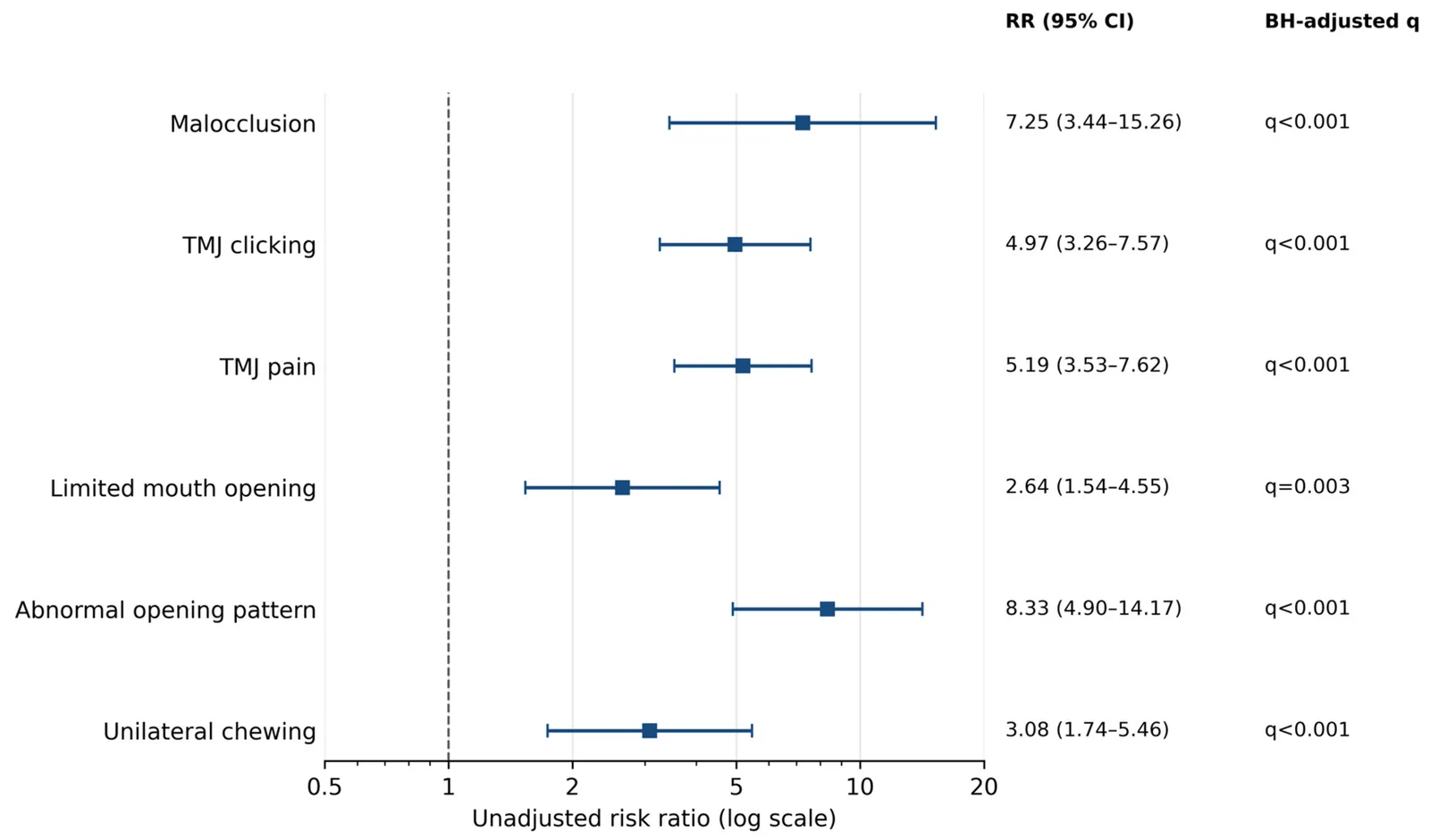
Unadjusted risk ratios for the six 3-month clinical findings that remained associated with clinician-recorded TMD at 12 months after multiplicity correction. Squares indicate RRs and horizontal bars indicate 95% CIs on a logarithmic scale. q values were obtained using Benjamini–Hochberg correction across all 11 testable exploratory variables. The complete family, including nonsignificant findings and baseline deep overbite, is reported in Supplementary Table S1. These estimates quantify longitudinal associations between overlapping clinical manifestations and should not be interpreted as independent prognostic effects or measures of prognostic performance. (The gray dashed vertical line indicates the null value of RR = 1.0).

Six 3-month clinical findings remained associated with the 12-month endpoint after Benjamini–Hochberg correction (Figure 4). Abnormal mouth-opening pattern had the largest point estimate (RR, 8.33; 95% CI, 4.90–14.17), followed by malocclusion (RR, 7.25; 95% CI, 3.44–15.26), TMJ pain (RR, 5.19; 95% CI, 3.53–7.62), TMJ clicking (RR, 4.97; 95% CI, 3.26–7.57), unilateral chewing (RR, 3.08; 95% CI, 1.74–5.46), and limited mouth opening (RR, 2.64; 95% CI, 1.54–4.55); all six q values were ≤0.003.

Postoperative anxiety did not retain evidence of association after multiplicity correction (RR, 2.22; 95% CI, 1.14–4.33; Fisher exact *p* = 0.061; q = 0.084). Infection, bruxism, and clenching were not associated with the endpoint. Deep overbite was recorded in four patients, all of whom had TMD at 12 months (RR, 4.03; 95% CI, 2.94–5.53; Fisher exact *p* = 0.005; q = 0.008); however, this was a baseline anatomical characteristic, and the estimate was based on only four exposed patients. Complete results for all 11 variables, including nonsignificant findings, are reported in Supplementary Table S1.

TMJ clicking, pain, limited opening, and abnormal opening trajectory are also manifestations considered during later TMD assessment. Their longitudinal associations may therefore represent persistence or recurrence of the same clinical construct rather than prediction of a biologically distinct future disorder. The dataset did not contain a standardized 3-month TMD diagnosis that would support a landmark analysis restricted to patients free of TMD at 3 months. These RRs quantify longitudinal associations between overlapping clinical manifestations; they do not evaluate discrimination, calibration, or independent prognostic performance.

## 4. Discussion

### 4.1. Principal Findings

This study evaluated a clinician-recorded treating-team endpoint rather than a formal DC/TMD diagnosis. In this retrospective cohort of 121 patients with an observed 12-month outcome after digitally planned mandibular fracture repair, clinician-recorded TMD was documented in 27.3%. The principal injury-specific finding was the concentration of the endpoint among patients with condylar involvement: 57.4% compared with 8.1% without condylar involvement. The association remained strong in a deliberately limited adjusted model and was insensitive to ordinal versus categorical representation of displacement grade. Broad fracture region was not associated with the endpoint, suggesting that recorded joint-specific anatomy was more informative than the general mandible-only, combined, or panfacial classification in this dataset.

The observed frequency should not be compared directly with general-population prevalence. A recent meta-analysis estimated global TMD prevalence at approximately 34%, but estimates vary by geography, age, and diagnostic method [9]. Older population studies have reported lower pain-focused estimates [15,16,17]. The present cohort comprised trauma patients without documented persistent preinjury TMD and used a treating-team clinical endpoint rather than a fully protocolized population survey. A prospective study of surgically treated non-condylar mandibular fractures illustrates how strongly the observed frequency depends on ascertainment: at 6 months, 12.9% had severe subjective symptoms whereas 80.6% had clinical dysfunction signs according to complementary Helkimo indices [10]. The present 27.3% is therefore best interpreted as a clinically substantial, center-specific frequency that justifies structured surveillance, not as a prevalence benchmark or evidence of excess risk relative to another treatment pathway.

### 4.2. Condylar Injury and TMD

The association with condylar injury is clinically plausible because the fracture directly affects the articulating segment, periarticular soft tissues, joint loading, and mandibular movement. Acute condylar injury can be accompanied by disk, capsular, and other soft-tissue abnormalities [7]. The highest observed TMD frequency followed condylar-head fractures, but the level-specific groups were small and their CIs overlapped; the data do not establish a stable hierarchy across condylar levels. Moreover, the dataset did not retain side-specific condylar treatment, open versus closed management, surgical approach, or fixation details. The observed association therefore combines effects of injury anatomy, severity, treatment selection, and postoperative care and should not be interpreted as an isolated anatomical effect.

Recent population-based evidence is directionally consistent but not directly comparable: a Swedish registry study found that prior mandibular fracture had the strongest association with subsequent clinically managed TMJ disorder among craniomaxillofacial injuries (adjusted odds ratio, 11.4) [18]. That study used registry diagnoses and treatment use rather than a protocolized post-fracture DC/TMD examination, so it supports the relevance of trauma history but does not validate the present 12-month frequency or effect size.

Treatment heterogeneity is also clinically relevant. A 2023 systematic review of condylar fracture management identified only four eligible comparative studies and found insufficient evidence to designate either surgical or nonsurgical management as uniformly reliable, although surgery permitted faster functional recovery in some settings and treatment selection was influenced by age, occlusion, and other clinical factors [19]. This limited and heterogeneous evidence reinforces that unrecorded treatment choice and rehabilitation could confound the present association rather than merely add random variability.

The single recorded mandibular-site variable was also associated with TMD, driven partly by the condylar category. However, 28 of 47 patients with condylar involvement had another site recorded in that single field, confirming that multi-site fractures had been compressed into one code. This variable is therefore descriptive and should not be interpreted as a complete anatomical classification or entered alongside condylar involvement without careful consideration. Future datasets should use separate binary indicators for each mandibular site and record left and right condylar levels independently.

### 4.3. Interpretation of Early Clinical Signs

Malocclusion showed a strong longitudinal association with the 12-month endpoint. Experimental occlusal interference can alter condylar position and movement trajectory [20]. Clinically, a newly unstable bite should prompt repeat occlusal and TMJ assessment. Nevertheless, the present data cannot determine whether malocclusion preceded TMJ dysfunction, resulted from altered jaw use, reflected treatment selection, or marked more severe injury.

TMJ clicking, pain, limited opening, and abnormal movement trajectories are clinically relevant findings, but they also overlap with the later endpoint definition. Joint noise and abnormal trajectories may reflect altered disk–condyle coordination, whereas pain and limited opening may reflect inflammation, mechanical loading, or protective muscle behavior. Arthrographic and electromyographic evidence links internal derangement and TMD to altered condylar paths or compensatory masticatory muscle behavior [21,22]. CBCT studies describe osseous degenerative changes in relation to joint space and demographic factors, whereas systematic MRI evidence more directly links effusion with pain [23,24]. Because systematic follow-up MRI and a standardized 3-month TMD diagnosis were unavailable, structural mechanisms and independent prognostic value cannot be inferred.

Unilateral chewing may reflect pain avoidance, occlusal discomfort, asymmetric muscular recovery, or learned compensation. The source data retained only a binary classification, not continuous chewing-laterality measurements. Collectively, the 3-month findings are best interpreted as correlates of persistence or recurrence that warrant reassessment; they do not support construction of a risk calculator or claims of independent prediction.

More standardized studies illustrate the information not available here. The OPPERA cohort used prospective, examiner-verified TMD ascertainment and collected psychosocial risk information when evaluating incident injury and subsequent TMD [8]. In Chinese TMD populations, the JFLS has demonstrated reliability and validity [25], and DC/TMD-based assessment has linked symptom burden with functional limitation [26]. After condylar fracture, combining objective masticatory performance with a validated patient-reported function measure has shown that objective performance and perceived ability can diverge [27]. By contrast, the present outcome collapses diagnosis, symptoms, and functional impact into a clinician-recorded label and lacks Axis II measures; direct prevalence and severity comparisons are therefore inappropriate.

### 4.4. Clinical Implications

The findings support a follow-up pathway that prioritizes patients with condylar injury while recognizing that the present study did not compare alternative pathways. Occlusal and TMJ symptom screening can begin at 1 month, followed by standardized measurement of maximal interincisal opening, movement trajectory, pain, clicking, and chewing laterality at 3 months. Consistent with recent INfORM/IADR good-practice points and evidence supporting complementary physical and psychosocial assessment [28,29], follow-up should, where resources allow, pair a calibrated Axis I examination with brief Axis II measures of pain-related disability, psychological distress, oral behaviors, and jaw functional limitation rather than symptom recording alone. Persistent abnormalities should prompt reassessment, rehabilitation review, and referral when appropriate. Prospective care pathways should record condylar treatment, fixation, the timing and content of physiotherapy, occlusal adjustment, splint therapy, medication, and secondary procedures.

Digital reconstruction should incorporate functional checkpoints in addition to bony alignment. Dental-arch scanning, virtual articulation when feasible, attention to condylar seating, and postoperative occlusal verification may help identify correctable problems. These are clinical considerations, not evidence that a specific digital component prevents TMD: all participants received a digital workflow, optional components were heterogeneous and incompletely recorded, and no conventional-treatment group was available. This caution is consistent with reviews describing broad variation in the applications and reporting of virtual planning and three-dimensional printing across oral and maxillofacial surgery [30].

### 4.5. Strengths and Limitations

Strengths include a defined 12-month interval, complete data among analyzed patients, explicit absolute and relative effect estimates, multiplicity correction, full reporting of all testable exploratory variables, and sensitivity analysis of displacement coding. The condylar association remained strong in a deliberately limited adjusted model. These strengths should be considered alongside several limitations.

First, the single-center retrospective design, modest sample, and absence of a conventional-treatment group preclude causal or comparative-effectiveness conclusions and increase susceptibility to selection bias. Eligibility required complete imaging records and assessable occlusion and excluded patients with severe systemic comorbidity or unreliable premorbid occlusal reference; these criteria improve outcome assessability but may preferentially select healthier, dentate patients with more complete documentation. The cohort is most comparable to adolescents and adults treated at tertiary maxillofacial centers using digitally assisted workflows and structured follow-up; generalizability is limited for children, older or medically complex patients, edentulous patients, and institutions with different operative protocols, rehabilitation strategies, referral thresholds, or access to digital planning. Second, the endpoint was a treating-team diagnosis without preserved item-level DC/TMD algorithms, a reproducible minimum case definition, subtype diagnoses, examiner calibration, systematic MRI, or documented blinding. Differential outcome ascertainment according to known injury morphology cannot be excluded. Third, preinjury TMD status depended partly on recall and available records, so mild or intermittent preinjury disease may have been missed.

Fourth, morphology variables were retrospectively abstracted without reconstructable duplicate masked review or interobserver reliability. Only one mandibular-site code was retained despite possible multi-site fractures; side-specific condylar level, quantitative displacement thresholds, and open-fracture definitions were unavailable. Subgroup estimates were based on small cells. Fifth, side-specific condylar management, open versus closed treatment, surgical approach, fixation details, standardized counts of optional digital components, rehabilitation adherence, and individualized treatment between 3 and 12 months were not retained. These factors may confound or modify the observed associations. Because treatment intensity and rehabilitation may have been selected in response to injury severity or early dysfunction, confounding by indication could inflate an association when complex care marks greater injury severity or attenuate it when targeted treatment reduces later symptoms; neither the direction nor the magnitude can be quantified from these data.

Sixth, 18 of 139 eligible patients (12.9%) were lost before the 12-month assessment, and their patient-level baseline data were unavailable. Extreme-case bounds describe only the possible range of the overall frequency and cannot quantify exposure-specific attrition bias. Loss to follow-up could have biased both frequency and association estimates if retention depended jointly on condylar injury and persistent symptoms. Seventh, several clinical variables were based on nonstandardized documentation, and the 3-month findings overlapped with manifestations used to determine the later endpoint. Finally, only 33 outcome events occurred, leaving subgroup estimates imprecise and limiting the number of parameters that could be estimated reliably. The adjusted analysis was therefore restricted to condylar involvement and three prespecified covariates; broader adjustment would likely have overfit the data, and the 3-month analyses remained unadjusted and exploratory.

Prospective multicenter studies should use calibrated and blinded DC/TMD Axis I examinations, include Axis II measures of pain-related disability, psychological distress, oral behaviors, and jaw functional limitation, and distinguish pain-related and intra-articular subtypes. They should combine validated patient-reported measures with objective functional testing, record all mandibular fracture sites and side-specific condylar levels, apply quantitative displacement definitions, and document condylar treatment, fixation, digital components, rehabilitation, and interventions during follow-up. A standardized 3-month TMD assessment would permit landmark analyses among patients free of TMD at that time. Larger samples could then support prespecified multivariable modeling, assessment of interactions, and internal validation.

## 5. Conclusions

Clinician-recorded TMD was documented in 27.3% of analyzed patients at 12 months after digitally planned mandibular fracture repair and was concentrated among those with condylar injury. The association persisted after limited adjustment and sensitivity analysis of displacement coding. Malocclusion, TMJ symptoms, restricted or abnormal jaw movement, and unilateral chewing at 3 months were longitudinally associated with the later endpoint, but several findings overlapped with TMD manifestations and may represent persistence or recurrence rather than independent prediction. The results support closer functional surveillance after condylar injury but do not establish causality, an isolated anatomical effect, or the comparative effectiveness of digital planning.

## Figures and Tables

**Table 1:** Demographic and injury characteristics by 12-month TMD outcome (*n* = 121).

Variable	Overall (*n* = 121)	TMD (*n* = 33)	No TMD (*n* = 88)	*p* Value
Age, years	39.59 ± 13.63	40.18 ± 13.53	39.36 ± 13.74	0.769
Male sex	84 (69.4%)	21 (63.6%)	63 (71.6%)	0.398
**Fracture region**				**0.339**
Mandible only	64 (52.9%)	14 (42.4%)	50 (56.8%)	
Combined maxillomandibular	35 (28.9%)	11 (33.3%)	24 (27.3%)	
Panfacial	22 (18.2%)	8 (24.2%)	14 (15.9%)	
**Fracture etiology**				**0.819**
Road traffic accident	45 (37.2%)	12 (36.4%)	33 (37.5%)	
Fall from height	18 (14.9%)	6 (18.2%)	12 (13.6%)	
Ground-level fall	58 (47.9%)	15 (45.5%)	43 (48.9%)	
Condylar involvement	47 (38.8%)	27 (81.8%)	20 (22.7%)	<0.001
**Recorded mandibular site**				**<0.001** ^†^
Symphysis/parasymphysis	27 (22.3%)	6 (18.2%)	21 (23.9%)	
Body	28 (23.1%)	6 (18.2%)	22 (25.0%)	
Angle	26 (21.5%)	2 (6.1%)	24 (27.3%)	
Ramus	15 (12.4%)	5 (15.2%)	10 (11.4%)	
Condyle	19 (15.7%)	13 (39.4%)	6 (6.8%)	
Coronoid	6 (5.0%)	1 (3.0%)	5 (5.7%)	
Bilateral fracture	51 (42.1%)	11 (33.3%)	40 (45.5%)	0.229
**Displacement grade**				**0.290**
No displacement	36 (29.8%)	7 (21.2%)	29 (33.0%)	
Mild displacement	39 (32.2%)	10 (30.3%)	29 (33.0%)	
Marked displacement	46 (38.0%)	16 (48.5%)	30 (34.1%)	
Comminuted fracture	21 (17.4%)	7 (21.2%)	14 (15.9%)	0.591
Open fracture	63 (52.1%)	15 (45.5%)	48 (54.5%)	0.418
Deep overbite (baseline)	4 (3.3%)	4 (12.1%)	0 (0.0%)	0.005

Data are mean ± standard deviation or *n* (%); percentages are within columns. Age: Welch *t* test. Pearson chi-square: sex, fracture region, etiology, laterality, and displacement grade; other testable binary variables: Fisher exact. ^†^ Monte Carlo permutation test (200,000 permutations); the single-site code is descriptive.

## Data Availability

De-identified data are available from the corresponding author upon reasonable request, subject to institutional and ethical restrictions.

## Supplementary Materials

The following supporting information can be downloaded at https://www.mdpi.com/article/10.3390/jcm15166426/s1, Table S1: Associations of recorded 3-month findings with clinician-recorded TMD at 12 months; Table S2: Modified-Poisson regression models for condylar involvement and clinician-recorded TMD at 12 months.
