# Supplementary material for: Clinician-Recorded Temporomandibular Disorder at 12 Months After Digitally Planned Mandibular Fracture Repair: Associations with Condylar Injury and Early Functional Signs
Source: J Clin Med. 2026 Aug 20;15(16):6426. doi: 10.3390/jcm15166426 (PMC13514207; doi:10.3390/jcm15166426)
Supplement: Supplementary file 1 [file jcm-15-06426-s001.zip › jcm-4480887-supplementary.pdf]

SUPPLEMENTARY MATERIAL

Clinician-recorded temporomandibular disorder at 12 months after digitally planned mandibular fracture repair: associations with condylar injury and early functional signs

Supplementary Table S1. Complete exploratory associations of recorded clinical characteristics with clinician-recorded TMD at 12 months (n = 121).

Characteristic	TMD with characteristic, n/N (%)	TMD without characteristic, n/N (%)	RR (95% CI)	Fisher P	BH-adjusted q
Limited mouth opening	15/29 (51.7%)	18/92 (19.6%)	2.64 (1.54–4.55)	0.001	0.003
TMJ clicking	13/14 (92.9%)	20/107 (18.7%)	4.97 (3.26–7.57)	<0.001	<0.001
TMJ pain	12/12 (100.0%)	21/109 (19.3%)	5.19 (3.53–7.62)	<0.001	<0.001
Abnormal opening pattern	21/21 (100.0%)	12/100 (12.0%)	8.33 (4.90–14.17)	<0.001	<0.001
Infection	1/3 (33.3%)	32/118 (27.1%)	1.23 (0.24–6.26)	1.000	1.000
Malocclusion	26/41 (63.4%)	7/80 (8.8%)	7.25 (3.44–15.26)	<0.001	<0.001
Bruxism	6/23 (26.1%)	27/98 (27.6%)	0.95 (0.44–2.02)	1.000	1.000
Unilateral chewing	19/37 (51.4%)	14/84 (16.7%)	3.08 (1.74–5.46)	<0.001	<0.001
Postoperative anxiety	5/9 (55.6%)	28/112 (25.0%)	2.22 (1.14–4.33)	0.061	0.084
Clenching	2/9 (22.2%)	31/112 (27.7%)	0.80 (0.23–2.83)	1.000	1.000
Non-union	0/0 (NE)	33/121 (27.3%)	NE	—	—
Deep overbite (baseline)	4/4 (100.0%)	29/117 (24.8%)	4.03 (2.94–5.53)	0.005	0.008

BH, Benjamini-Hochberg; CI, confidence interval; NE, not estimable; RR, risk ratio; TMJ, temporomandibular joint. Values are TMD events/total patients and risk percentages within the characteristic-present and characteristic-absent groups. All 11 testable exploratory variables, including nonsignificant findings, are reported. Non-union is shown for reporting completeness but was not testable and was excluded from the multiplicity family because it was absent in all 121 patients. Deep overbite was included in the correction family but is a baseline anatomical characteristic and should be interpreted separately. Two-sided Fisher exact P values were adjusted using the Benjamini-Hochberg procedure. These unadjusted estimates quantify associations; several 3-month findings overlap with manifestations used for the 12-month endpoint and should not be interpreted as independent prognostic effects or measures of prognostic performance.

Supplementary Table S2. Modified-Poisson regression estimates for the association of condylar involvement with clinician-recorded TMD at 12 months (n = 121; 33 outcome events).

Variable	Primary model, RR (95% CI)	P	Categorical-displacement sensitivity model, RR (95% CI)	P
Condylar involvement	6.87 (3.06–15.41)	<0.001	7.34 (3.41–15.78)	<0.001
Age, per 10 years	1.02 (0.86–1.20)	0.844	1.02 (0.86–1.21)	0.797
Male sex	0.83 (0.51–1.35)	0.454	0.83 (0.50–1.38)	0.479
Displacement grade, per category	1.21 (0.93–1.59)	0.160	—	—
Mild displacement vs none	—	—	1.82 (0.88–3.77)	0.107
Marked displacement vs none	—	—	1.59 (0.85–2.98)	0.147

CI, confidence interval; RR, risk ratio. Both models used a log link, Poisson working variance, and HC0 robust sandwich covariance; P values are two-sided Wald tests. The primary model included condylar involvement, age per 10 years, male sex, and displacement grade coded ordinally (none, mild, marked). The sensitivity model replaced the ordinal term with separate indicators for mild and marked displacement, using no displacement as the reference. Covariates were selected before outcome modeling. With only 33 events, open-fracture status, comminution, and additional injury variables were not added because the extra parameter burden could cause overfitting and unstable estimates. These parsimonious models estimate explanatory associations and are not exhaustive adjustment or prediction models; post-treatment 3-month clinical findings were not included.
